# Impact of Grain Sorghum Polyphenols on Microbiota of Normal Weight and Overweight/Obese Subjects during In Vitro Fecal Fermentation

**DOI:** 10.3390/nu11020217

**Published:** 2019-01-22

**Authors:** Danielle Ashley, Daya Marasini, Cindi Brownmiller, Jung Ae Lee, Franck Carbonero, Sun-Ok Lee

**Affiliations:** 1 Department of Food Science, Division of Agriculture, University of Arkansas, Fayetteville, AR 72704, USA; dmbenedi@email.uark.edu (D.A.); marasini@uark.edu (D.M.); cbrownm@uark.edu (C.B.); fgcarbon@uark.edu (F.C.); 2 Agricultural Statistics Laboratory, Division of Agriculture, University of Arkansas, Fayetteville, AR 72704, USA; julee@uark.edu

**Keywords:** sorghum bran polyphenols, gut microbiota, sumac sorghum bran, black sorghum bran, short-chain fatty acids

## Abstract

The human gut microbiota is considered as a crucial mediator between diet and gut homeostasis and body weight. The unique polyphenolic profile of sorghum bran may promote gastrointestinal health by modulating the microbiota. This study evaluated gut microbiota and modulation of short-chain fatty acids (SCFA) by sorghum bran polyphenols in in vitro batch fermentation derived from normal weight (NW, *n* = 11) and overweight/obese (OO, *n* = 11) subjects’ fecal samples. Six separate treatments were applied on each batch fermentation: negative control (NC), fructooligosaccharides (FOS), black sorghum bran extract (BSE), sumac sorghum bran extract (SSE), FOS + BSE, or FOS + SSE; and samples were collected before and after 24 h. No significant differences in total and individual SCFA production were observed between NW and OO subjects. Differential responses to treatment according to weight class were observed in both phyla and genera. Sorghum bran polyphenols worked with FOS to enhance *Bifidobacterium* and *Lactobacillus*, and independently stimulated *Roseburia* and *Prevotella* (*p* < 0.05). Our results indicate that sorghum bran polyphenols have differential effects on gut health and may positively impact gut ecology, with responses varying depending on weight class.

## 1. Introduction

In the past decades, the human colon has come to light as a hub for microbial activities that impact many aspects of human health. The gut microbiota is composed of hundreds to thousands of species with biological activities including nutrient metabolism, vitamin synthesis, immune modulation, pro- and anti-microbial activities, and modulation of gut homeostasis [1]. Dysbiosis, or microbial imbalance, has been implicated in a number of chronic progressive conditions, one of which is obesity. More than 70% of adults in the United States are classified as overweight or obese; not only do these conditions threaten quality of life, but they also increase the risk of chronic diseases such as type 2 diabetes, heart disease, and stroke [2,3]. Evidence from animal and human studies indicates a role of the gut microbiota in body weight maintenance and development of obesity, although exact mechanisms remain unclear [4]. Marked differences in bacterial population have been seen in the microbiota of overweight/obese individuals when compared to normal weight individuals [5,6,7,8]. These findings implicate the gut microbiota as a potential target of nutritional therapies to prevent/reduce weight gain and obesity.

While most food consumed is digested and absorbed in the stomach and small intestine, complex carbohydrates such as resistant starch and fiber reach the colon where they are utilized by the gut microbiota [9]. These compounds are fermented by certain species to produce short-chain fatty acids (SCFA) which have numerous health implications including body weight maintenance [8,10,11,12]. Prebiotics were previously defined as “selectively fermented ingredient(s) that result in specific changes in the composition and/or activity of the gastrointestinal microbiota, thus conferring benefit(s) upon host health” and the compounds that fit these criteria were limited to mainly inulin, galactooligosaccharides (GOS), and fructooligosaccharides (FOS) [13]. In 2017 however, prebiotics were redefined as “a substrate that is selectively utilized by host microorganisms conferring a health benefit” [14]. This new definition may allow for inclusion of non-carbohydrate foods that do not undergo fermentation but are utilized by beneficial species in the gut, thus enhancing their expansion and positive health benefits. Such compounds include polyphenols.

Polyphenols are biologically active compounds produced by metabolic pathways in plants, with numerous roles including pathogen protection, antimicrobial, and antioxidant activities [15]. Most polyphenols consumed by humans are polymerized and/or glycosylate. These high molecular weight compounds reach the colon where they are broken down by the gut microbiota to smaller, absorbable compounds [16]. Grain sorghum (*Sorghum bicolor* L. Moench) commonly called sorghum, is the world’s fifth highly produced cereal crop, far behind the top four (rice, corn, wheat, and barley) but commonly grown in specific areas. Sorghum contains various classes of polyphenols, which are in the bran fraction [17]. The polyphenol composition of sorghum bran varies with its color, with sumac varieties rich in proanthocyanidins and black varieties rich in 3-deoxyanthocyanins, which include luteolinidin and apigeninidin [18]. Though previous studies have identified positive modulatory effects of polyphenols from polyphenol-rich foods on the human gut microbiota [19,20,21,22,23,24], to our knowledge no research has been conducted with sorghum polyphenol extracts. The objectives of this research were to characterize the major polyphenol components of two sorghum brans, and to evaluate the change of gut microbiota composition and the effect on SCFA production in response to sorghum bran polyphenols in fecal samples from normal weight (NW) and overweight/obese (OO) subjects.

## 2. Materials and Methods

### 2.1. Sorghum Brans, Standards, and Reagents

Black and sumac sorghum brans were purchased from local market and Nu Life Market (Scott City, KS, USA) and fructooligosaccharides (FOS) from Megazyme International Ireland Ltd. (Wicklow, Ireland). Folin–Ciocalteu’s reagent, 4-dimethylaminocinnamaldehyde (DMAC), 2,2- diphenyl-1-picrylhydrazyl (DPPH), butyric acid, propionic acid, and acetic acid were obtained from Sigma-Aldrich (St. Louis, MO, USA). Luteolinidin and apigeninidin were from Chromadex (Irvine, CA, USA).

### 2.2. Preparation of Polyphenols from Sorghum Bran Samples

Polyphenol extractions were performed according to Awika et al. [18] with modifications. Defatted sorghum bran samples (5 g) and extracting solvent (1% HCl in 70% methanol) were mixed at a ratio of 1:60. Mixtures were stirred at room temperature for 2 h and filtered through Whatman No. 42 filter paper (GE Healthcare, Amersham, UK). Solvent was removed using rotary evaporation at 30 °C and the aqueous filtrate applied to a pre-activated C-18 solid phase extraction (SPE) column (Grace Davidson Discovery Sciences, Deerfield, IL, USA). Sugars were eluted with 100 mL of water and discarded; then the remaining polyphenols were eluted with 100 mL of methanol. After elution of polyphenols, 50 mL of deionized water were then added to methanolic extract and methanol was removed through rotary evaporation at 30 °C. The concentrated aqueous extract was then frozen and subsequently lyophilized in a VirTis Benchtop SLC freeze dryer (SP Industries, Warminster, PA, USA). Resulting powdered polyphenol extracts, black sorghum bran extract (BSE), and sumac sorghum bran extract (SSE), were then pooled and stored in a desiccator at −4 °C until further analysis. Dried polyphenol extracts were used as substrates in the in vitro fecal fermentation experiment.

### 2.3. Analysis of Polyphenol Content and Antioxidant Activity

Total polyphenol content of sorghum bran extracts (BSE and SSE) was determined using the Folin–Ciocalteu assay according to Singleton and Rossi [25] with minor modifications. Standards were prepared using gallic acid in concentrations of 3.125, 6.25, 12.5, 25, 50, and 100 μg/g. BSE and SSE were dissolved for a dilution of 8000-fold. In a 48-well plate, 0.1 mL sample or standard, 0.5 mL of 0.2 N Folin–Ciocalteu’s reagent, and 0.4 mL of 7.5% Na_2_CO_3_ were combined. The plate was incubated at room temperature for 2 h and the absorbance at 760 nm was determined in a Synergy TT microplate reader (BioTek Instruments, Winooski, VT, USA). All samples and standards were measured in triplicate. Concentration was determined by plotting against a gallic acid standard curve and results were thus expressed in gallic acid equivalents (GAE).

Total proanthocyanidin content was determined using the 4-dimethylaminocinnamaldehyde (DMAC) assay [26]. Standards were prepared using catechin and ethanol (95%) at concentrations of 0.125, 6.25, 12.5, 25, 50, and 100 μg/g. In a 48-well plate, 0.15 mL of sample (BSE and SSE in ethanol for a dilution of 8000-fold) or standard and 0.75 mL of 5.7 N DMAC solution were combined, all in at least triplicate. Plates were read immediately at 640 nm in microplate reader. Concentration was determined by plotting against a catechin standard curve and expressed in catechin equivalents.

For quantitative analysis of 3-deoxyanthocyanins (3-DXA) a Beckman Coulter (Fullerton, CA, USA) System Gold HPLC system was used. The system was equipped with a 126 pump, a 168 Detector, and a 508 autosampler. The software used for data collection and integration was 32 Karat 8. A 250 × 4.6 mm i.d. C18 column (YMC America Inc, Allentown, PA, USA) was used for separation of 3-DXA in sorghum and standards. The mobile phase consisted of A: 5% formic acid in water, and B: 100% methanol. Flow rate was 1 mL min^−1^ and injection volume was 30 µL. The mobile phase gradient of Cho et al. [27] was used, with minor modifications: 0–60 min: 2–60% B, 60–61 min: 60–2% B, 61–66 min: 2% B, 66–75 min: 2–0% B. Wavelengths monitored were 280, 340, and 480 nm. Apigeninidin and luteolinidin were identified and quantified using standard curves.

Antioxidant properties were determined using the free radical 2,2- diphenyl-1-picrylhydrazyl (DPPH) assay according to Brand-Williams et al. [28]. DPPH solution was prepared by dissolving in methanol (0.15 mM) and stored at −20 °C between uses. Serial dilutions of BSE and SSE (1–140 ppm) were prepared in methanol. DPPH solution (0.95 mL) was added to 0.05 mL extracts of varying concentrations and shaken. Plates were read in microplate reader at 30 min at 517 nm. Effective concentration to reduce the radical by 50% (EC_50_) was determined for both bran extracts and reported as µg gallic acid equivalents per gram. All samples and standards were plated in at least triplicate.

### 2.4. Human Fecal Fermentation

After approval of the study by the International Review Board (University of Arkansas; IRB #17-02-433), 22 subjects were recruited from the North West Arkansas area: 11 normal weight (NW, body mass index (BMI) < 25) and 11 overweight/obese (OO, BMI ≥ 25) subjects. Exclusion criteria included tobacco use, digestive disease, fasting blood glucose (FBG ≥ 100 mg/dL), current medications, and antibiotic use within the past six months. Consent and screening forms as well as food frequency questionnaires were filled out during screening sessions. FFQ (Food Frequency Questionnaire) responses were analyzed using Axxya System Nutritionist Pro™ software version 4.3.0 (Stafford, TX, USA) based on USDA References. No significant differences in macro- and micro-nutrient intake were observed between weight class groups or genders. Participant characteristics are provided in Table 1. Selected participants were given a stool collection kit and delivered samples within an hour of defecation. Fecal samples were transferred to anaerobic chamber immediately for fermentation experiment.

Fecal fermentation medium was prepared according to Yang et al. [29]. One liter of anerobic fermentation medium was composed of yeast extract (2 g; Alfa Aesar, Ward Hill, MA, USA), peptone (2 g; Fisher Scientific, Waltham, WA, USA), bile salts (0.5 g; Oxoid, Hampshire, UK), NaHCO_3_ (2 g), NaCl (0.1 g), K_2_HPO_4_ (0.08 g), MgSO_4_.7H_2_O (0.01 g), CaCl_2_∙6H_2_O (0.01 g), L-cysteine hydrochloride (0.5 g; Sigma, St. Louis, MO, USA), vitamin K (10 μL; Sigma, St. Louis, MO, USA), bovine hemin (50 mg; Sigma, St. Louis, MO, USA), Tween 80 (2 mL), and 0.025% (*w*/*v*) resazurin solution. To prepare fecal slurry, 2 g fecal sample was added to 20 mL phosphate-buffered saline, vortexed to homogenization, and filtered through four layers of cotton gauze. After the analyses of total polyphenol content, the amount of BSE and SSE was calculated taking into account the grape extract and black tea extract (0.6–1 g/L). Within an anaerobic chamber, 14 mL of sterile fermentation medium were inoculated with 1 mL of fecal slurry and treated with either no substrate (negative control, NC), FOS, BSE, SSE, FOS + BSE, and FOS + SSE. The final concentration of FOS and grain sorghum bran extract (BSE and SSE) was 5 g/L and 1 g/L, respectively. Test tubes were incubated at 37 °C and aliquots (~3 mL) taken at the time points 0, 6, 12, 18, and 24 h. Aliquots were added to 0.2 mL of 2 M KOH stop solution and stored at −80 °C until further analysis.

### 2.5. Short-Chain Fatty Acid (SCFA) Analysis

Short-chain fatty acids (SCFA) content was determined for all samples at all time point using the method of Bourquin et al. [30] with modification. After thawing at room temperature, 450 µL of samples were combined with 50 µL of a prepared solution containing 50 g meta-phosphoric acid and 1.6 g CuSO_4_/L, and 314.6 μL 4-methyl valeric acid (internal standard). After incubation at room temperature for 10 min, the mixture was centrifuged at 11,500 rpm for 5 min and the supernatant collected for analysis. SCFA analysis was performed using a Varian CP-3800 GC (Agilent, Santa Clara, CA, USA) with a CP-8400 autosampler and a HP-FFAP (High Polarity for the analysis of Free Fatty Acids and Phenols) modified polyethylene glycol (25 m × 32 mm) column. One µL of sample was injected with a split of 30:1 and a flow rate of 1.3 mL/min. The gradient used was 3 °C/min from 65 °C to 110 °C and 8 °C/min until 150 °C. Total and individual SCFA were quantified against reference standards for butyric acid (BA), propionic acid (PA), and acetic acid (AA).

### 2.6. Microbial Analysis

Changes of microbiota profiles in response to treatments were determined by analyzing bacterial DNA of samples from time point 0 and 24 h following protocols previously described [31]. DNA was extracted using the QIAamp Fast DNA Stool Mini Kit (Qiagen, Gaithersburg, MD, USA). The V4 region of the bacterial 16S-rRNA gene was amplified by polymerase chain reaction (PCR) in Eppendorf Mastercycler Pro S (Eppendorf, Hamburg, Germany) using AccuPrime Pfx SuperMix and V4 index primers [32]. After confirming the amplification through agarose gel electrophoresis, DNA samples were normalized with SequalPrep Normalization Plate Kit (Thermo Fisher Scientific, Waltham, WA, USA). The concentration of final pooled library of the V4 region of 16S-rRNA gene of bacteria was sequenced utilizing the Illumina MiSeq platform [32]. Fastq files generated through Illumina were demultiplexed and quality filtered, operational taxonomic units (OTUs) assigned using SILVA database, and the data processed through Mothur 1.40.1 [33].

### 2.7. Experimental Design and Statistical Analysis

Each of the 22 subjects received all six treatments (NC, FOS, BSE, SSE, FOS + BSE, FOS + SSE). Therefore, the statistical analysis was performed for the randomized complete block design (RCB), in which the effect of six treatments was tested with subjects as blocks. We note that the analysis of variance (ANOVA) approach for RCB seriously violated the assumptions of normality and equal variance in our diagnostic procedures. Alternatively, Friedman test was used, known as a distribution-free rank sum test for RCB, followed by pairwise comparisons among six treatments at different time points: 6, 12, 18, 24 h for SCFA data and 0, 24 h for microbiota data, respectively. The time effect (0 vs. 24 h) was tested using a Wilcoxon signed rank sum test, as an alternative to a paired *t*-test, if there was a significant increase/decrease in relative abundance among 22 subjects. The effect of weight class (NW vs. OO with 11 subjects each) was tested using a Mann–Whitney test, as an alternative to two-sample *t*-test, under different treatments. All tests were nonparametric to avoid any controversial assumption for small sample size. The false discovery rate adjustment for the *p*-value was conducted as appropriate. Non-metric multidimensional scaling (NMDS) was utilized to analyze similarities in bacterial communities between treatments. PAST 3.15 was used for NMDS and analysis of similarities (ANOSIM) according to the Bray–Curtis index. The statistical results were reported at 0.05 or 0.01 levels of significance. All data analyses were conducted using statistical software SPSS (IBM, Armonk, NY, USA) and R version 3.3.3 (R core Team, Vienna, Austria).

## 3. Results

### 3.1. Polyphenol Profile and Antioxidant Properties of Sorghum Bran Extracts

Total polyphenols and proanthocyanidins were significantly higher in sumac sorghum bran extract (SSE), and 3-deoxyanthocyanins in black sorghum bran extract (*p* < 0.01) (Table 2). Radical scavenging capability was assessed using the DPPH radical, and both black (BSE, 274.0 ± 20.3 µg gallic acid equivalent (GAE)/g) and sumac (SSE, 269.5 ± 21.0 µg GAE/g) sorghum bran extracts demonstrated antioxidant capabilities with no significant differences between the two.

### 3.2. Short-Chain Fatty Acid Production

**Figure 1:**
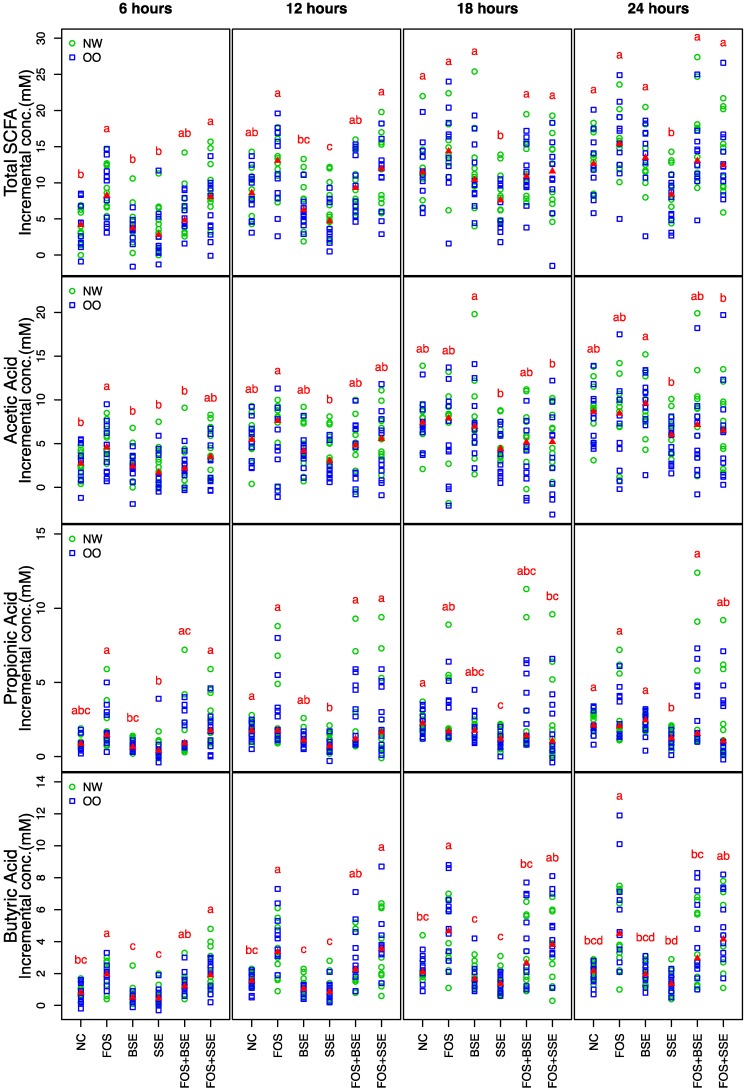
Total and individual SCFA during fermentation by treatment in both normal weight (NW, *n* = 11) and overweight/obese (OO, *n* = 11) samples. Different letters indicate significant differences between treatments, achieved by the Friedman test and its post hoc multiple comparisons at 0.05 level of significance. The medians are marked with red triangles. The weight classes (NW vs. OO) are labeled by green circles and blue squares, respectively. NC: negative control, FOS: fructooligosaccharides, BSE: black sorghum bran extract, SSE: sumac sorghum bran extract; SCFA: short-chain fatty acids.

No significant differences in total and individual SCFA production was observed between NW and OO. Total SCFA concentration was significantly increased by FOS and FOS + SSE compared to the NC, BSE, and SSE at 6 h (*p* < 0.05) (Figure 1). SSE resulted in lower levels of total SCFA from time points 12 h to 24 h (Figure 1).

FOS alone increased acetic acid (AA) compared to NC, BSE, SSE, and FOS + BSE at 6 h, and SSE displayed lower concentrations compared to FOS at 12 h (*p* < 0.05) (Figure 1). BSE increased AA concentration compared to SSE and FOS + SSE at time points of 18 h and 24 h (*p* < 0.05).

Incremental propionic acid (PA) concentration was generally higher in NW than OO, though not significantly. SSE caused lower concentrations of PA at 12, 18, and 24 h compared to NC (*p* < 0.05) (Figure 1).

Incremental butyric acid (BA) concentrations in FOS and FOS + SSE treatments were significantly higher than NC, BSE, and SSE at 6 h and 12 h (*p* < 0.05) (Figure 1). FOS increased BA compared to NC, BSE, and SSE at all time points (*p* < 0.05). Sorghum extracts alone did not alter BA concentrations. 

### 3.3. General Changes in the Microbiota

**Figure 2:**
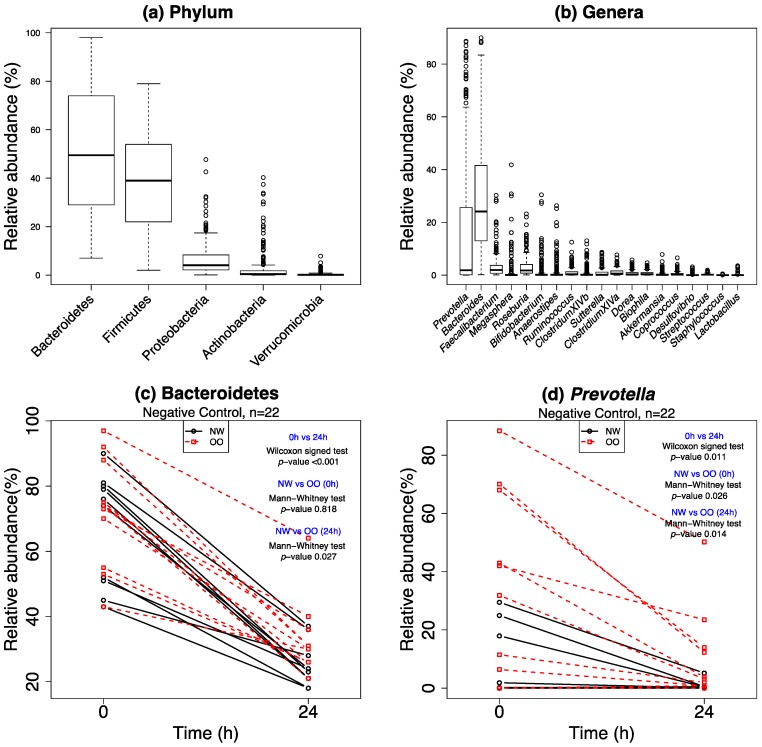
Dynamics of microbiota (phylum and genera level) in in vitro fermentation. In (**a**,**b**), the distribution of microbiota is depicted in the boxplot sorted by largest variance. In (**c**,**d**), the time effect, 0 h vs. 24 h, is shown and tested by a Wilcoxon signed test, and the group difference between NW and OO is tested by a Mann–Whitney test under the negative control (*n* = 22) for two selected microbiota.

After DNA extraction and sequencing of 264 samples, a total of 5,062,231 high quality reads were obtained for analysis of microbial populations. Overall distribution of microbiota, the comparison between NW and OO, and a change between time point 0 h and 24 h are shown in Figure 2. At the phylum level, the fermentation was dominated by Bacteroidetes, in which we observed a significant drop over the past 24 h, a significant difference between NW and OO at 24 h, and an insignificant difference between NW and OO at 0 h under no treatment (negative control, bottom left panel). A similar interpretation is possible for all microbiota in both phylum and genera. This analysis narrows down our focus on certain microbiota, and shows how we stratified the situations for weight class and time effect to be tested. The results for all microbiota are summarized in Table 3 and Table 4.

**Figure 3:**
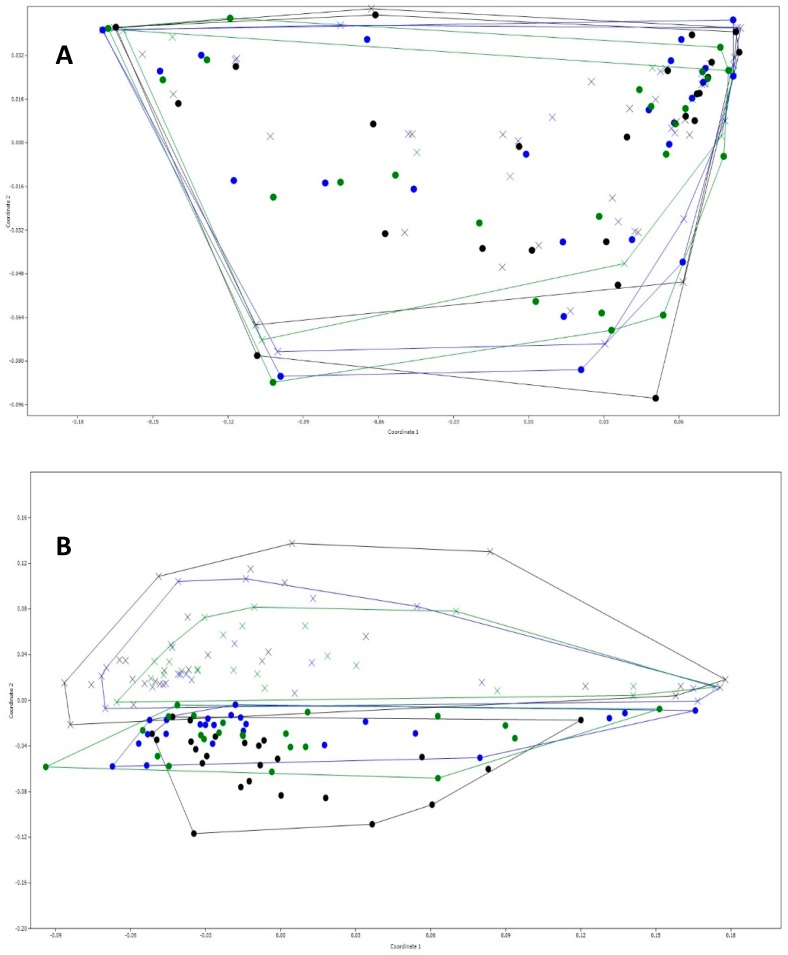
Non-metric multidimensional scaling (NMDS) plot comparing bacterial communities between treatments in both groups combined (*p* < 0.05). (**A**) 0 h and (**B**) 24 h. ●: negative control (NC), **x**: fructooligosaccharides (FOS), ●: black sorghum bran extract (BSE), ●: sumac sorghum bran extract (SSE), **x**: FOS + BSE, **x**: FOS + SSE.

Non-metric multidimensional scaling (NMDS) plots revealed no differences in bacterial communities between treatments at time point 0 (Figure 3A), however after 24 h a clear separation was seen between the NC and FOS-containing treatments (*p* < 0.05) (Figure 3B). Sorghum extracts alone did not significantly impact bacterial communities compared to the negative control. 

### 3.4. Specific Changes in Microbial Population

**Figure 4:**
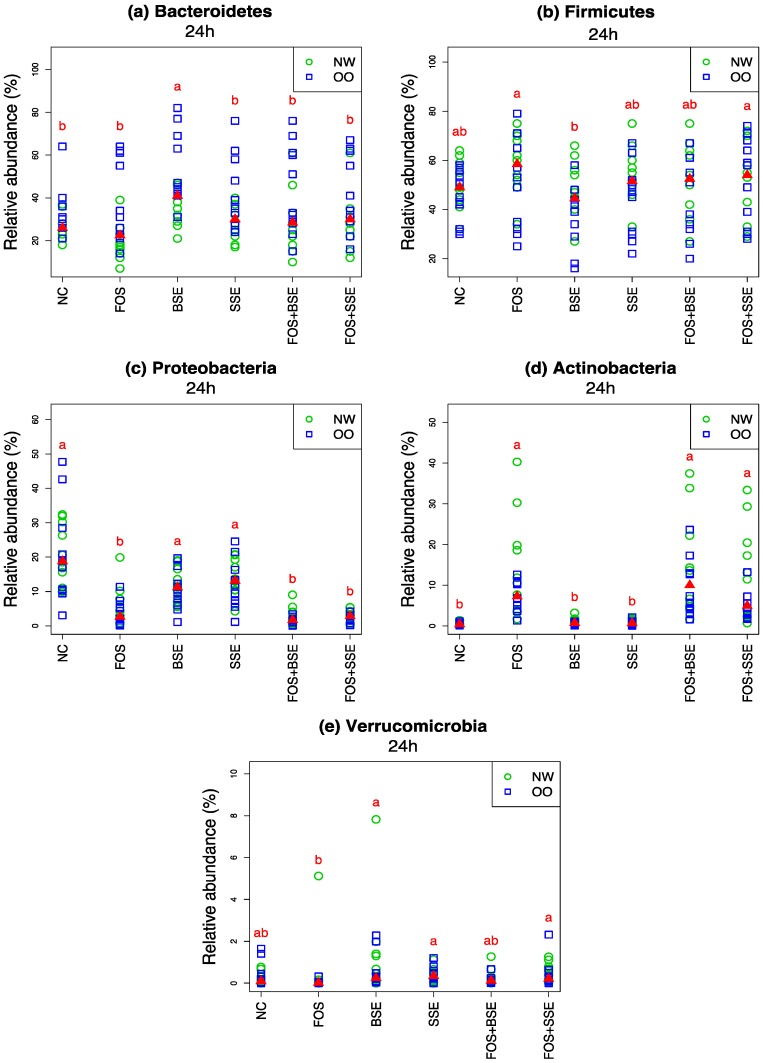
Relative abundance at phylum level (*n* = 22). (**a**) Bacteroidetes, (**b**) Firmicutes, (**c**) Proteobacteria, (**d**) Actinobacteria, (**e**) Verrucomicrobia. Letters indicate significant differences between treatments at 24 h, achieved by the Friedman test and its post hoc multiple comparisons at the 0.05 level of significance. The medians are marked with red triangles. The weight classes (NW vs. OO) are labeled by green circles and blue squares, respectively. NC: negative control, FOS: fructooligosaccharides, BSE: black sorghum bran extract, SSE: sumac sorghum bran extract.

After 24 h of in vitro fermentation, the OO group displayed higher abundance of Bacteroidetes than the NW group (Figure 4). We observed relative decrease in Bacteroidetes in all treatments (Table 3). In all subjects BSE resulted in significantly higher abundance of Bacteroidetes at 24 h than NC and all other treatments (*p* < 0.05) (Figure 4).

As relative abundance of Bacteroidetes decreased there was a corresponding increase in Firmicutes (Table 3). BSE had significantly lower abundance of Firmicutes at 24 h compared to FOS and FOS + SSE (*p* < 0.05) (Figure 4).

FOS, FOS + BSE, and FOS + SSE had significantly lower abundance of Proteobacteria after 24 h compared to NC, BSE, and SSE (*p* < 0.05) (Figure 4).

NW tended to display a greater response of Actinobacteria than OO in FOS, FOS + BSE and FOS + SSE (Figure 4). While NC, BSE, and SSE showed negligible increases of Actinobacteria after 24 h, FOS, FOS + BSE and FOS + SSE increased at significantly higher magnitudes of 10%, 12%, and 9%, respectively (*p* < 0.05) (Table 3).

Compared to FOS, relative abundance of Verrucomicrobia increased in BSE, SSE and FOS + SSE after 24 h of in vitro fermentation (*p* < 0.05) (Figure 4).

**Figure 5:**
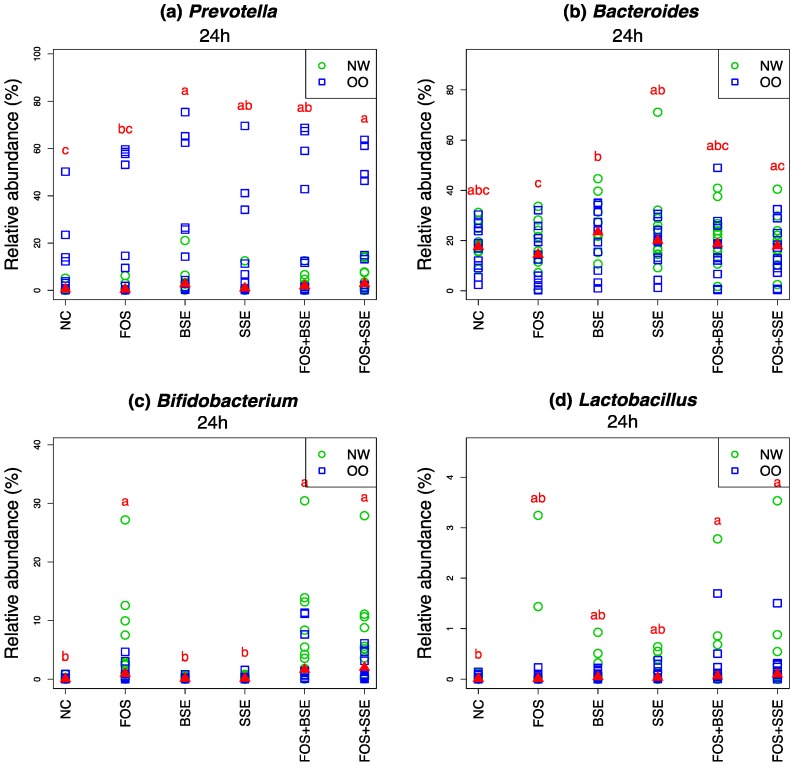
Relative abundance of select genera (*n* = 22). (**a**) *Prevotella*, (**b**) *Bacteroides*, (**c**) *Bifidobacterium*, (**d**) *Lactobacillus*. Letters indicate significant differences between treatments at 24 h, achieved by the Friedman test and its post hoc multiple comparisons at 0.05 level of significance. The medians are marked with red triangles. The weight classes (NW vs. OO) are labeled by green circles and blue squares, respectively. NC: negative control, FOS: fructooligosaccharides, BSE: black sorghum bran extract, SSE: sumac sorghum bran extract.

*Prevotella* was present at higher levels in OO than NW in all treatments after 24 h (Figure 5). Relative abundance of *Prevotella* decreased about 15% in the NC but to a lesser extent in all treatments (Table 4). At 24 h, abundance was significantly higher than the NC for BSE, SSE, FOS + BSE and FOS + SSE (*p* < 0.05) but not for FOS alone (Figure 5). Additionally, BSE and FOS + SSE had significant higher levels than FOS (*p* < 0.05) (Figure 5).

Though *Bacteroides* tended to be higher in NW samples, no significant differences were observed between the two groups after 24 h of in vitro fermentation. Both BSE and SSE resulted in higher abundance of *Bacteroides* than FOS (*p* < 0.05) (Figure 5). 

Samples from NW subjects tended to have higher levels of *Bifidobacterium* after 24 h than OO samples when treated with FOS and FOS plus sorghum polyphenols (Figure 5). *Bifidobacterium* relative abundance in FOS, FOS + BSE, and FOS + SSE was significantly higher than the NC, BSE, and SSE after 24 h (*p* < 0.05) (Figure 5). Sorghum polyphenols alone had no effect.

There were no significant differences between weight class groups for *Lactobacillus*. While *Lactobacillus* relative abundance was stable in the NC after 24 h, increases of this genus, though small, were seen in all treatments (Table 4). While FOS alone did not significantly increase abundance at 24 h compared to the NC, FOS + BSE and FOS + SSE had significantly higher levels compared to the NC (*p* < 0.05) (Figure 5). Sorghum polyphenols alone did not significantly affect *Lactobacillus*.

**Figure 6:**
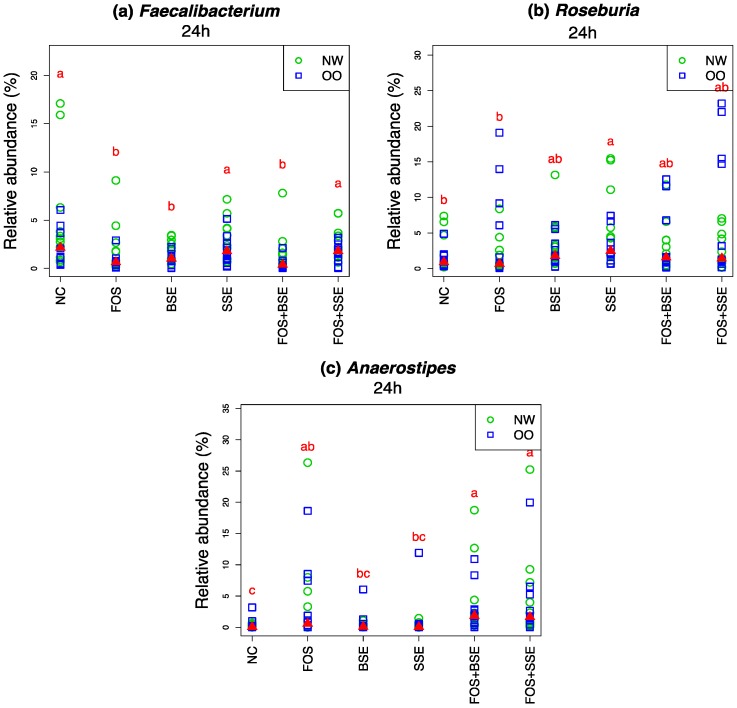
Relative abundance of butyric acid-producing bacteria (*n* = 22). (**a**) *Faecalibacterium*, (**b**) *Roseburia*, (**c**) *Anaerostipes*. Letters indicate significant differences between treatments at 24 h, achieved by the Friedman test and its post hoc multiple comparisons at the 0.05 level of significance. The medians are marked with red triangles. The weight classes (NW vs. OO) are labeled by green circles and blue squares, respectively. NC: negative control, FOS: fructooligosaccharides, BSE: black sorghum bran extract, SSE: sumac sorghum bran extract.

Abundance of *Faecalibacterium* tended to be higher at 24 h in NW than OO (Figure 6). There was little change in *Faecalibacterium* over 24 h in the NC (Table 4), however abundance decreased in response to all treatments and was significantly lower in FOS, BSE, and FOS + BSE at 24 h compared to NC, SSE, and FOS + SSE (*p* < 0.05) (Figure 6).

*Roseburia* was stimulated by sumac sorghum polyphenols, with SSE resulting in higher abundance than NC and FOS at 24 h (*p* < 0.05) (Figure 6).

No significant differences were seen in *Anaerostipes* between the two weight groups. Relative abundance of *Anaerostipes* was higher in FOS, FOS + BSE, and FOS + SSE compared to NC at 24 h (*p* < 0.05) (Figure 6). FOS + BSE and FOS + SSE significantly increased *Anaerostipes* compared to NC, BSE and SSE (*p* < 0.05) (Figure 6).

## 4. Discussion

As metabolic conditions such as obesity continue to burden large proportions of the U.S. population, the topic of gut health, and more specifically the gut microbiota, has gained increasing attention in the realms of health and nutrition. For this reason, a recent focus of nutrition research has been identifying whole foods as well as bioactive components with the potential to positively shift dysregulated bacterial populations towards more desirable profiles [34]. Of particular allure are plant polyphenols, as they are widely spread in nature and have been credited with the ability to positively modulate the gut microbiota. Not only is grain sorghum bran is a source of polyphenols, but it is also cost effectively and efficiently produced in the United States, making it an attractive candidate for nutraceutical applications.

Our current study found both black and sumac sorghum brans to contain various classes of polyphenols. In sorghum bran analysis, our findings of 27.5 ± 1.5 mg/g for black and 43.0 ± 2.0 mg/g for sumac are consistent with previous reports of total phenolics ranging from 7.6–35.6 mg/g and 22.5–88.5 mg/g for black and sumac sorghum bran, respectively [17,18,35]. The two varieties had strikingly distinct phenolic profiles, with higher concentrations of 3-Deoxyanthocyanins (3-DXA) in black bran, and higher concentrations of proanthocyanidins in sumac bran. These results are in agreement with multiple previous analyses of black and sumac sorghum, in which black sorghum is established as enriched in 3-DXA, and sumac in proanthocyanidins [17,18,36,37].

It has been well established that grain sorghum bran has significant antioxidant capabilities [17,18,36,37,38,39], and our current analyses sought to compare the radical scavenging properties of the two different varieties of sorghum bran extract. Despite lower levels of total polyphenols in black sorghum bran extract (BSE), both BSE and sumac sorghum bran extract (SSE) showed similar radical scavenging capabilities.

Fermentation with the wine/grape and black tea polyphenols resulted in significantly increased acetate concentrations compared to blank in a study by Gross et al. [40]. However, in the present study, the levels of acetate and butyrate did not change during the fermentations with BSE and SSE compared to NC. Propionate production was lower in SSE compared to the NC, which is not in agreement with the report of Gross et al. [40] who found no change of propionate production with the wine/grape and tea polyphenols. FOS-containing treatments in this study increased butyrate production over 24 h. *Lactobacillus* and *Bifidobacterium* are both known to utilize FOS to produce lactic acid [41], which can be further metabolized to butyrate by genera such at *Anaerostipes* and *Eubacterium* [42]. We found increases in *Bifidobacterium*, *Lactobacillus*, and *Anaerostipes* in response to FOS-containing treatments, so it is plausible that the increases in butyrate were due to these cross-feeding pathways. This is one of the first studies to examine the impact of sorghum bran polyphenols on SCFA production and further work is needed to corroborate these findings. 

Although NMDS did not reveal a significant impact of sorghum polyphenols on the overall microbial communities, the sorghum extracts modulated the gut microbiota at both phyla and genus levels, and combined sorghum polyphenols and FOS worked to enhance specific beneficial genera. Our data showed relative shifts in the two major phyla, Firmicutes increasing in abundance and Bacteroidetes decreasing. This is not surprising, as many carbohydrate utilizers, including the probiotic *Lactobacillus*, are within Firmicutes, and the conditions of the experiment promote utilization of these substrates, including those already present in the fecal samples.

Proteobacteria contains several potentially pathogenic bacteria [43] avoiding the overgrowth of this phylum may be a positive outcome of nutrition strategies. Lower abundance of Proteobacteria likely reflect the increases in groups that utilize FOS. Though not statistically significant, BSE and SSE caused a marked decrease in Proteobacteria compared to the NC, suggesting they may act antagonistically against some pathogenic species. These results are similar to those of Pham et al. [44], who found that during in vitro fecal fermentation Proteobacteria was decreased by FOS, feruloylated arabinoxylans (FAXO), proanthocyanidin-rich rice bran polyphenols, and FAXO and rice bran polyphenols combined.

*Prevotella* is often associated with long-term high carbohydrate diets [45]. *Prevotella* was stimulated but there were no significant differences between sorghum polyphenols with and without FOS. A study with 62 obese subjects found that individuals with a high *Prevotella:Bacteroides* ratio lost significantly more weight in response to a high-fiber diet than individuals with lower levels of *Prevotella* [46]. Although further research is required to reach a deeper understanding of this interaction, enhancing the ratio of *Prevotella:Bacteroides* may assist in weight-loss strategies that are based on increased fiber intake. In the present study, OO samples resulted in increased *Prevotella:Bacteroides* with higher levels of *Prevotella* after 24 h.

Though health-promoting bacterial genera in the colon are not limited to *Lactobacillus* and *Bifidobacterium*, they are the traditional targets of prebiotic supplementation [14]. *Bifidobacterium* proliferation was enhanced by FOS-containing treatments. Compared to the NC, FOS + BSE and FOS + SSE tended to increase abundance of *Bifidobacterium* in OO. This data suggests utilization of FOS by *Bifidobacterium* is altered in OO microbiota, and that sorghum polyphenols may enhance fermentation of FOS by this species in OO subjects. As decreased proportions of *Bifidobacterium* have been seen previously in overweight/obese subjects [6,7], nutritional therapies to increase this genus may be extremely beneficial to individuals combatting excessive body weight gain. Past in vitro fermentation studies have observed stimulation of *Bifidobacterium* by polyphenols such as the anthocyanin malvidin-3-glucoside [47], tart cherries [31], and grape and red wine polyphenols [23,48]. In our experiment, however, this genus was not apparently impacted by sorghum polyphenols alone.

In the present study, FOS and sorghum polyphenols worked synergistically to enhance *Lactobacillus*. As *Lactobacillus* was present at low abundance, further studies are needed to corroborate these effects. Success of prebiotic research in stimulating growth of *Lactobacillus* has been markedly lower than with *Bifidobacterium* [14], and the ability of polyphenols to enhance oligosaccharide utilization by *Lactobacillus* would provide a new avenue of prebiotic supplementation. As human clinical trials have attributed several strains of *Lactobacillus* with anti-obesogenic actions [49], this mode of supplementation may be especially beneficial in body weight maintenance. In addition, previous studies have observed stimulation of *Lactobacillus* by red wine polyphenols, grape seed extract monomers, and anthocyanin malvidin-3-glucoside [19,47,48].

Additional targets of prebiotic supplementation include butyrate-producing bacteria. Butyrate is not only the main energy source of colonocytes, but research has purported numerous roles in colon-cancer antagonism, inflammation suppression, and colonic barrier function [50]. In our study, FOS-containing treatments increased butyrate production and this trend was paralleled by increased abundance of *Anaerostipes*. *Anaerostipes* produces butyrate by metabolizing lactate produced by other species or utilizing acetate through the butyryl CoA: acetate CoA transferase pathway [51], and our results suggest that this genus was responsible for much of the butyrate production during this in vitro fermentation.

*Roseburia* is a genus with the ability to produce butyrate through the butyryl CoA: acetate CoA transferase pathway [52]. Though little research has been done with this genus, reduced abundance of *Roseburia* has been a marker of dysbiosis in both ulcerative colitis [53] and colorectal cancer [54]. Previous studies have identified stimulation of *Roseburia* by various carbohydrate sources [55,56], but this was not significantly impacted by FOS-containing treatments in the present study. SSE, on the other hand, caused significant increases compared to both NC and FOS, indicating utilization of sumac sorghum polyphenols by *Roseburia*. This is not the first report of *Roseburia* stimulation by polyphenols, as this genus also increased in response to red rice bran polyphenols [44].

## 5. Conclusions

Black and sumac sorghum brans are significant sources of polyphenols with diverse polyphenol profiles. Although sorghum extracts did not significantly influence SCFA production from FOS, they enhanced proliferation of *Prevotella* and the butyrate-producing genus *Roseburia*. Combined sorghum polyphenols and FOS worked to enhance *Bifidobacterium* and especially *Lactobacillus*, a probiotic genus that has been difficult to stimulate through prebiotic supplementation. We observed differential responses to treatment in NW and OO microbiota for *Prevotella*, *Bifidobacterium*, and *Roseburia*, supporting the theory that gut microbial metabolism is altered in OO. Our results indicate that sorghum bran polyphenols may help modulate gut microbial populations, especially in concert with other prebiotic substances such as FOS. Further research is needed to determine which individual polyphenol compound is responsible for beneficial effects on the gut microbiota and to understand the mechanism by which the microbial strains respond to polyphenols.

## Figures and Tables

**Table 1:** Subject characteristics

	All (*n* = 22)	Normal Weight		Overweight/Obese	
		Male (*n* = 5)	Female (*n* = 6)	Male (*n* = 5)	Female (*n* = 6)
Age (years)	29.0 ± 6.7	29.4 ± 4.8	24.2 ± 2.1	28.2 ± 3.5	34.2 ± 9.7
BMI (kg/m^2^)	27.1 ± 5.5	23.0 ± 0.7	22.2 ± 1.2	30.9 ± 2.9	32.2 ± 5.4
FBG (mg/dL)	92.1 ± 4.9	92.9 ± 3.1	90.6 ± 5.0	92.6 ± 6.9	96.3 ± 3.3

Data are expressed as mean ± standard deviation (SD). BMI: body mass index, FBG: fasting blood glucose.

**Table 2:** Major polyphenol profile of black and sumac sorghum bran extracts.

	BSE	SSE
Polyphenols (mg gallic acid equiv/g extract)	321.5 ± 2.7	571.7 ± 6.0 *
Proanthocyanidins (mg catechin equiv/g extract)	8.4 ± 1.4	86.9 ± 1.1 *
3-Deoxyanthocyanins (mg/g extract)	10.1 ± 0.3 *	2.0 ± 0.4
Luteolinidin	9.3 ± 0.4 *	2.0 ± 0.4
Apigeninidin	0.9 ± 0.1 *	ND

Data are expressed as mean ± standard deviation (SD). * Indicates significant difference between black and suman bran extracts. *p* < 0.01. *n* = 8. BSE: black sorghum bran extract, SSE: sumac sorghum bran extract, ND: not detected.

**Table 3:** The effects of time and weight class in dominant phyla.

Microbiota	Treatment	Change in 24 h Relative Abundance (%)	Wilcoxon Signed Test (0 h vs. 24 h) *q*-Value †	Mann-Whitney Test (NW vs. OO) at 24 h *q*-Value †
Bacteroidetes	NC	−41.10	<0.001	0.136
Bacteroidetes	FOS	−43.50	<0.001	0.085
Bacteroidetes	BSE	−23.60	<0.001	0.085
Bacteroidetes	SSE	−31.00	<0.001	0.085
Bacteroidetes	FOS + BSE	−35.10	<0.001	0.177
Bacteroidetes	FOS + SSE	−32.10	<0.001	0.246
Firmicutes	NC	22.40	0.001	0.491
Firmicutes	FOS	32.00	<0.001	0.489
Firmicutes	BSE	14.00	0.006	0.107
Firmicutes	SSE	19.70	<0.001	0.180
Firmicutes	FOS + BSE	24.10	<0.001	0.491
Firmicutes	FOS + SSE	23.80	0.002	0.974
Proteobacteria	NC	17.40	<0.001	0.640
Proteobacteria	FOS	1.00	1.000	0.489
Proteobacteria	BSE	7.70	<0.001	0.491
Proteobacteria	SSE	10.00	<0.001	0.491
Proteobacteria	FOS + BSE	−1.40	0.034	0.180
Proteobacteria	FOS + SSE	−1.20	0.058	0.190
Actinobacteria	NC	0.30	0.003	0.825
Actinobacteria	FOS	10.30	<0.001	0.491
Actinobacteria	BSE	0.70	<0.001	0.061
Actinobacteria	SSE	0.60	<0.001	0.180
Actinobacteria	FOS + BSE	11.80	<0.001	0.280
Actinobacteria	FOS + SSE	9.20	<0.001	0.180
Verrucomicrobia	NC	−0.10	0.867	0.926
Verrucomicrobia	FOS	−0.10	0.076	0.636
Verrucomicrobia	BSE	0.60	0.079	0.489
Verrucomicrobia	SSE	0.00	0.463	0.926
Verrucomicrobia	FOS + BSE	−0.10	0.949	0.378
Verrucomicrobia	FOS + SSE	0.10	0.215	0.491

† adjusted *p*-value by false discovery rate. A change in relative abundance over 24 h was tested using a Wilcoxon signed test. A Mann–Whitney test was used to test the difference between NW (normal weight) vs. OO (overweight/obesity) at 24 h under each treatment. NC: negative control, FOS: fructooligosaccharides, BSE: black sorghum bran extract, SSE: sumac sorghum bran extract.

**Table 4:** The effects of time and weight class at the genus level.

Microbiota	Treatment	Change in 24 h Relative Abundance (%)	Wilcoxon Signed Test (0 h vs. 24 h) *q*-Value †	Mann-Whitney Test (NW vs. OO) at 24 h *q*-Value †
*Prevotella*	NC	−14.50	0.022	0.557
*Prevotella*	FOS	−8.00	0.010	0.557
*Prevotella*	BSE	−7.10	0.145	0.557
*Prevotella*	SSE	−11.90	0.017	0.557
*Prevotella*	FOS + BSE	−7.60	0.087	0.639
*Prevotella*	FOS + SSE	−7.00	0.057	0.639
*Bacteroides*	NC	−23.90	0.001	0.779
*Bacteroides*	FOS	−27.90	<0.001	0.727
*Bacteroides*	BSE	−14.60	0.019	0.800
*Bacteroides*	SSE	−14.40	0.028	0.755
*Bacteroides*	FOS + BSE	−22.50	0.001	0.721
*Bacteroides*	FOS + SSE	−21.40	0.003	0.719
*Bifidobacterium*	NC	0.10	0.059	0.875
*Bifidobacterium*	FOS	3.50	0.001	0.579
*Bifidobacterium*	BSE	0.10	0.018	0.755
*Bifidobacterium*	SSE	0.20	0.016	0.589
*Bifidobacterium*	FOS + BSE	5.30	<0.001	0.638
*Bifidobacterium*	FOS + SSE	4.30	<0.001	0.557
*Lactobacillus*	NC	0.00	0.919	0.721
*Lactobacillus*	FOS	0.20	0.300	0.957
*Lactobacillus*	BSE	0.10	0.059	0.782
*Lactobacillus*	SSE	0.10	0.025	0.721
*Lactobacillus*	FOS + BSE	0.30	0.011	0.755
*Lactobacillus*	FOS + SSE	0.30	0.004	1
*Faecalibacterium*	NC	−0.40	0.314	0.579
*Faecalibacterium*	FOS	−2.10	0.007	0.721
*Faecalibacterium*	BSE	−3.90	0.001	0.579
*Faecalibacterium*	SSE	−3.10	0.017	0.684
*Faecalibacterium*	FOS + BSE	−3.50	<0.001	0.557
*Faecalibacterium*	FOS + SSE	−2.90	0.145	0.651
*Roseburia*	NC	−0.40	0.251	0.876
*Roseburia*	FOS	1.00	0.991	0.930
*Roseburia*	BSE	0.10	0.893	0.755
*Roseburia*	SSE	1.20	0.278	0.649
*Roseburia*	FOS + BSE	1.40	0.341	0.972
*Roseburia*	FOS + SSE	2.10	0.327	0.800
*Anaerostipes*	NC	0.30	0.047	0.755
*Anaerostipes*	FOS	3.80	0.001	0.972
*Anaerostipes*	BSE	0.40	0.077	0.755
*Anaerostipes*	SSE	0.70	0.057	0.779
*Anaerostipes*	FOS + BSE	3.40	<0.001	0.957
*Anaerostipes*	FOS + SSE	4.10	<0.001	1

† adjusted *p*-value by false discovery rate. A change in relative abundance over 24 h was tested using a Wilcoxon signed test. A Mann–Whitney test was used to test the difference between NW (normal weight) vs. OO (overweight/obesity) at 24 h under each treatment. NC: negative control, FOS: fructooligosaccharides, BSE: black sorghum bran extract, SSE: sumac sorghum bran extract.
